# Age-associated genes in human mammary gland drive human breast cancer progression

**DOI:** 10.1186/s13058-020-01299-2

**Published:** 2020-06-15

**Authors:** Xiang Gu, Bingzhi Wang, Haiyan Zhu, You Zhou, Aaron M. Horning, Tim H-M Huang, Yidong Chen, Peter Houghton, Zhao Lai, Joel E. Michalek, Lu-Zhe Sun

**Affiliations:** 1 Department of Cell Systems & Anatomy, School of Medicine, University of Texas Health Science Center at San Antonio, San Antonio, TX USA; 2 Xiangya Hospital and Xiangya School of Medicine, Central South University, Changsha, Hunan China; 3 Shanghai First Maternity and Infant Hospital, Tongji University School of Medicine, Shanghai, 200126 China; 4 Department of Molecular Medicine, School of Medicine, University of Texas Health Science Center at San Antonio, San Antonio, TX USA; 5 Department of Epidemiology & Biostatistics, School of Medicine, University of Texas Health Science Center at San Antonio, San Antonio, TX USA; 6 Greehey Children’s Cancer Research Institute, School of Medicine, University of Texas Health Science Center at San Antonio, San Antonio, TX USA; 7 Mays Cancer Center, School of Medicine, University of Texas Health Science Center at San Antonio, San Antonio, TX USA

**Keywords:** Aging, Breast cancer, Transcriptomics, Gene expression, Relapse-free survival, Tumor progression, DYNLT3, P4HA3, ALX4

## Abstract

**Background:**

Aging is a comorbidity of breast cancer suggesting that aging-associated transcriptome changes may promote breast cancer progression. However, the mechanism underlying the age effect on breast cancer remains poorly understood.

**Method:**

We analyzed transcriptomics of the matched normal breast tissues from the 82 breast cancer patients in The Cancer Genome Atlas (TCGA) dataset with linear regression for genes with age-associated expression that are not associated with menopause. We also analyzed differentially expressed genes between the paired tumor and non-tumor breast tissues in TCGA for the identification of age and breast cancer (ABC)-associated genes. A few of these genes were selected for further investigation of their malignancy-regulating activities with in vitro and in vivo assays.

**Results:**

We identified 148 upregulated and 189 downregulated genes during aging. Overlapping of tumor-associated genes between normal and tumor tissues with age-dependent genes resulted in 14 upregulated and 24 downregulated genes that were both age and breast cancer associated. These genes are predictive in relapse-free survival, indicative of their potential tumor promoting or suppressive functions, respectively. Knockdown of two upregulated genes (DYNLT3 and P4HA3) or overexpression of the downregulated ALX4 significantly reduced breast cancer cell proliferation, migration, and clonogenicity. Moreover, knockdown of P4HA3 reduced growth and metastasis whereas overexpression of ALX4 inhibited the growth of xenografted breast cancer cells in mice.

**Conclusion:**

Our study suggests that transcriptome alterations during aging may contribute to breast tumorigenesis. DYNLT3, P4HA3, and ALX4 play significant roles in breast cancer progression.

## Background

In addition to being a major risk for breast cancer, age was also shown to be associated with increased mortality in women with breast cancer [1]. The positive correlation between age and breast cancer incidence and mortality is believed to be due to the progressive accumulation of genetic and epigenetic alterations in breast epithelial cells as well as epigenetic changes in their microenvironment, which lead to changes of gene expression. However, the mechanism underlying the age effect on breast cancer remains poorly understood.

The genomic and epigenetic data of human adjacent normal tissues and tumor tissues, from public databases including The Cancer Genome Atlas (TCGA), have facilitated numerous findings in various cancers. Because age is a major risk for cancer and epigenetic alteration is one of hallmarks for both cancer and aging [2–4], it appears logical that if the expression of a gene is upregulated during aging and higher in tumor tissues than in their matched adjacent normal tissues, it is likely oncogenic. Conversely, if its expression is downregulated during aging and lower in tumors than their matched adjacent normal tissues, it is likely tumor-suppressive. Following this logic, we should be able to identify the genes associated with both age and breast cancer.

However, whether these in silico-identified age- and cancer-associated genes contribute to cancer development and progression, or whether they are no more than accidental correlation as passengers instead of drivers, remains inconclusive from statistical data mining. To answer these questions, experiments, where potential tumor-promoting genes are knocked down and potential tumor-suppressive genes are overexpressed in various in vitro and in vivo models, are needed to validate their direct effect on cancer progression.

In this study, we identified 38 novel age- and breast cancer-associated genes, which we call them ABC genes, by mining TCGA breast cancer data and validating using GTEx normal breast tissue data. We selected two upregulated and two downregulated ABC genes with significant prognostic values for various subtypes of breast cancer and further demonstrated that the upregulated ABC genes, DYNLT3 and P4HA3, have a tumor-promoting function whereas the downregulated gene ALX4, but not WDR86, showed tumor-suppressive function by in vitro and in vivo experiments. As such, our study provides novel molecular links between aging and breast cancer development and progression.

## Materials and methods

### Processing of TCGA RNAseq data

The RSEM estimated raw read counts data were downloaded from TCGA Data Portal on July 25, 2014, and normalized by library sizes, log2 transformed, and further normalized by quantile normalization. Removal of 25% of genes with the lowest mean expressions resulted in subsequent analyses on 15,398 genes. In identifying genes associated with age (birth_days_to), the genes whose expression were considered to be correlated with age were selected by three criteria: (1) *R*^2^ value among the highest 5%, (2) absolute value of slope for age among the highest 5%, and (3) adjusted *P* value for the slope among the lowest 5%. In identifying genes differentially expressed comparing post-menopausal normal against pre-menopausal normal, and comparing matched tumors against match normal samples, a combination of methods using moderate hierarchical *t* test (R/limma) and moderate fitting based on negative binomial distribution (R/DESeq2), with and without surrogate variable analysis for potential confounders (R/SVA) with cutoff as FDR < 0.05, were implemented. The common overlapping genes from four methods were determined as the final list for differentially expressed genes.

### Correlation between gene expression and subtypes of breast cancer

Two metrics were implemented to test the correlation between gene expression pattern of the 38 ABC genes and subtypes of breast cancer. The first metric was the sum of gene expression levels of 14 upregulated genes minus the sum of gene expression of 24 downregulated genes.

$$ \mathrm{Score}=\sum \limits_{\mathrm{g}\;\mathrm{in}\kern0.17em \mathrm{genes}}\mathrm{Expression}\left(\mathrm{g}\right)\times \mathrm{Direction}\left(\mathrm{g}\right) $$

The second metric was Pearson’s correlation coefficient between expression levels of 38 genes with their expression direction, 1 for upregulated and − 1 for downregulated genes.

*Score* = Pearson  Correlation (Expressions, Directions)

Both metrics were calculated for each patient then both metrics from all patients were examined against their subtypes.

### Gene set enrichment analysis

Normalized log2 expression data from TCGA breast cancer cohort were rank-ordered by fold change between any two subtypes. The signature comprised two sets, one for 14 upregulated genes and the other one for 24 downregulated genes. Genes in the expression data were not collapsed but directly compared with the signature. Gene signatures were tested using default enrichment GSEA statistics with 1000 random permutations.

### Survival analyses

Survival analyses, including disease/progression-free survival, were performed with “survival” package from R for patients in TCGA cohort. The split ratio to group patients into high and low groups based on gene expression was searched from 30% quantile to 70% quantile, and the best quantile which yields the lowest Cox regression *P* value was used as the best split ratio. To determine the prognostic value, Cox regression adjusted by diagnosis age was performed on two groups determined by the best split ratio.

For relapse-free survival on patients with aggregated microarray data, kmplot.com was used [5]. “Auto select best cutoff” was checked, and “only JetSet best probe set” was used to choose probeset for each queried gene. For the analysis on panel of genes, the weighted mean expression of all probeset was used to split patients.

For survival analysis of each individual gene, analyses were conducted on all patients and patients stratified by subtypes, in both TCGA RNASeq cohort and kmplot.com microarray cohort. For analysis on a panel of genes, analyses were conducted on all patients.

### Validation on GTEx dataset

The age information in unit of year of all donors in the GTEx database (www.gtexportal.org/home/) was applied and downloaded via dbGaP-controlled access. Among them, RNAseq gene counts data from 90 donors with normal breast tissue donation were extracted. This expression data was then normalized by library sizes, log2 transformed, and further normalized by quantile normalization. The 38 ABC genes identified from TCGA dataset were then queried in the normalized GTEx data, by studying the correlation between the gene expression and age. Multi-testing was addressed by Benjamini-Hochberg correction.

### Cell lines and culture conditions

The human breast cancer cell lines (MDA-MB-231, MDA-MB-468, ZR-75-1, SK-BR-3, Hs 578T, and BT-474) were purchased from the American Type Culture Collection (ATCC, Manassas, VA, USA), and MCF-7 cells were obtained from the Michigan Cancer Foundation in 1994. They were cultured in McCoy’s 5A medium supplemented with sodium pyruvate, amino acids, vitamins, penicillin, streptomycin, sodium bicarbonate, and 10% fetal bovine serum (FBS) in a humidified atmosphere containing 5% CO_2_ at 37 °C as previously described. These cells were authenticated in late 2016 with short tandem repeat assay by the DNA Lab in our university. They were routinely tested to be mycoplasma-free at least once a year with a luminescence-based mycoplasma detection kit from Lonza (Cat. # LT07-118).

### Transfection of siRNAs and screening

Three siRNAs per gene were purchased from Sigma-Aldrich. A scrambled siRNA (Sigma-Aldrich) was used as a negative control. Dried siRNAs were reconstituted with DEPC-treated water (10 μM Stock). Lipofectamine RNAiMAX reagent (Invitrogen) was diluted by adding 0.3 to 25 μl basic medium in each well. siRNAs (0.75 μl at 10 μM) were diluted in 25 μl basic medium and then mixed with suspended cells to reach 50 nM working concentration. Diluted RNAiMAX and siRNAs were incubated for 20 min at 37 °C. Cell suspension in 100 μl containing 3000 MDA-MB-231 cells, 3000 MDA-MB-468 cells, 7000 MCF7 cells, 5000 ZR-75-1 cells, 7000 SK-BR-3 cells, or 9000 Hs 578T cells in culture medium with 15% FBS were added to pre-incubated transfection reagent. Cell proliferation was monitored, and confluency was analyzed every 3 h in IncuCyte for 1 week.

### Molecular cloning and lentiviral vectors

MISSION® TRC2 pLKO.5-puro plasmids containing shRNAs against DYNLT3 and P4HA3 and MISSION® TRC2 pLKO.5-puro Empty Vector Control Plasmid were purchased from Sigma-Aldrich. MISSION® TRC2 pLKO.5-puro Empty Vector Control Plasmid was used as a control for these knockdowns. Plasmids harboring full-length CDS of ALX4 (HsCD00294887) and WDR86 (HsCD00436836) were purchased from DNASU Plasmid Repository (dnasu.org). Full-length CDS of ALX4 and WDR86 were then cloned by PCR with primers. The plasmid pWPI (addgene: 12254) was inserted at PmeI site with linker harboring BamHI, MluI, and NdeI sites, denoted as pLenti-Control. The cloned CDS of ALX4 and WDR86 were then inserted into plasmid pLenti-Control linearized by BamHI and PmeI, to construct pLenti-ALX4 and pLenti-WDR86 correspondingly. The plasmid pLenti-Control was used as a control for the overexpression.

### Viral production and infection of cell lines

293T packaging cells were transfected lentiviral vectors and packing vectors to generate lentiviral shRNAs (DYNLT3, P4HA3, and control) or CDS (ALX4, WDR86, and control) to infect MDA-MB-231 and BT-474 cells. Knockdown and overexpression were then examined with quantitative RT-PCR and Western blot analyses.

### RNA isolation and quantitative RT-PCR

Cells were lysed with QiaGen RLT Lysis buffer, and total RNAs were extracted using RNeasy Mini Kit (QiaGen: Cat No./ID: 74104) following the manufacturer’s protocol. cDNA was generated using random primers and M-MLV reverse transcriptase from Invitrogen (Grand Island, NY), and real-time PCR was performed using SYBR reagent (Invitrogen) as described before [6].

### Western blot analysis

Whole cell lysates were obtained in Laemmli buffer containing protease inhibitors and processed as described previously [6]. Antibody to DYNLT3 (Abcam, catalog # ab121209) was diluted 1:200 in 3% milk, to P4HA3 (Abcam, catalog # ab101657) was diluted 1:500 in 3% milk, to ALX4 (Biologicals, catalog # NBP2-45490) was diluted 1:1000 in 3% milk, to WDR86 (Thermo Fisher, catalog # PA5-48389) was diluted 1:1000 in 3% milk, to GAPDH (Calbiochem, catalog # 80602-840) was diluted 1:2500 in 3% BSA, and to tubulin (Sigma, Catalog # T4026) was diluted 1:5000 in TBST.

### Cell viability assay

Knockdown of DYNLT3 and P4HA3 or overexpression of ALX4 and WDR86 in MDA-MB-231 and BT-474, and corresponding knockdown or overexpression control cells were plated in at least triplicate wells at 2000 MDA-MB-231 cells or 5000 BT-474 cells per well into 96-well plates. In the following 1 week, MTT solution (50 μl, 2 mg/ml in PBS) was added to each well and allowed to incubate for 2 to 4 h in a cell culture incubator. After removing the culture medium, 100 μl DMSO was then added into each well with the plates gently agitated on a shaker. The absorbance was measured at 595 nm with a microplate reader (BioTek Instrument, Winooski, VT), which is proportional to the number of viable cells.

### Transwell migration assay

Cells were seeded at a density of 20,000 cells per well for MDA-MB-231 and 80,000 cells per well for BT-474 in serum-free medium in the upper 24-well Boyden chambers with 8-μm-size inserts (BD Biosciences, Durham, NC, USA). A serum-containing medium was added to the lower chamber. After allowing the cells to migrate for 16 to 18 h in cell culture incubator at 37 °C, the cells on the top of the membrane were removed and the migrated cells were fixed and stained with HEMA 3 STAIN SET from the Fisher Scientific Company. Picture of the stained cells on the insert was captured under a microscope at × 4 magnification for counting [7].

### Soft agar assay

Soft agar assays were carried out as described previously [7]. Growth of cells in soft agar was determined by plating 10,000 cells in triplicate in 0.3% Noble agar in 6-well tissue culture plates. Three weeks after plating, soft agar plates were stained and counted as described previously [7].

### Xenograft experiment in mice

Animal experiments were conducted following appropriate guidelines. They were approved by the Institutional Animal Care and Use Committee and monitored by the Department of Laboratory Animal Resources at the University of Texas Health Science Center at San Antonio. Five million exponentially growing luciferase- and GFP-expressing BT-474 control or DYNLT3 knockdown, or P4HA3 knockdown cells in 0.1 ml volume were injected into the left inguinal mammary fat pad of ten 6-week-old female NOD *scid* gamma (NSG) mice purchased from The Jackson Laboratory. Similarly, nine 6-week-old female NSG mice were inoculated with 5 × 10^6^ exponentially growing luciferase- and GFP-expressing BT-474 control or ALX4 OE, or WDR86 OE cells. About 10 days after inoculation, tumor size was recorded twice a week. Tumor volumes were calculated as tumor volume (mm^3^) = length × width × width/2. For the detection and quantification of lung metastasis biofluorescence imaging with IVIS, mice were euthanized at the termination of the experiment. d-luciferin (200 μl at 15 mg/ml) was injected intraperitoneally. Ten minutes later, the lung tissues from each mouse were excised and transferred to wells of a 24-well plate and were immediately imaged to obtain photo flux. Living image software was used for analysis. Tumor tissues were then excised, weighted, and cut into pieces to be frozen at − 80 °C or fixed in 10% formalin for various analyses.

### Statistics and reproducibility

Results are represented as described in the figure legends, with mean ± sem (standard error of mean). For the experiments with two groups and normally distributed data, the difference of means was tested for significance with two-tailed unpaired Student’s *t* test with the assumption of unequal variance. For multiple independent groups, one-way ANOVA was used. All experiments were repeated at least three times, and representative images and figures were shown.

## Result

### Identification of ABC genes

To identify genes with age-dependent expression, we extracted whole transcriptome profiling data of tumor-matched adjacent normal tissues from 82 female patients with age at diagnosis and menopausal status available in TCGA cohort (Supplementary Figure 1A). We applied simple linear regression to study the association between the gene expression level and age at diagnosis on the matched adjacent normal samples from all 82 patients, and from 54 post-menopausal patients to reduce the leverage effect of pre-menopausal patients with limited age range. We identified 210 upregulated and 98 downregulated genes in all patients (Supplementary Figure 1B), as well as 103 upregulated and 164 downregulated genes in post-menopausal patients only with the selection criteria defined in the “Materials and methods” section (Supplementary Figure 1C). The unions of these genes (258 upregulated (Supplementary Figure 1D) and 240 downregulated (Supplementary Figure 1E)) were considered as genes affected by age. Many of these genes were also influenced by menopausal status. Therefore, by comparing post-menopausal to pre-menopausal patients, we identified 493 upregulated (Supplementary Figure 1F) and 254 downregulated (Supplementary Figure 1G) genes altered by menopause. Exclusion of these menopause affected genes from those genes affected by age (258 upregulated and 240 downregulated) resulted in 148 upregulated (Supplementary Figure 1H) and 189 downregulated (Supplementary Figure 1I) genes during aging. Next, by comparing the matched tumors from the 82 patients against their matched adjacent normal samples, we identified 3356 upregulated (Supplementary Figure 1J) and 3124 downregulated (Supplementary Figure 1K) genes in breast cancer. The overlapping between these breast cancer-associated genes and age-associated genes resulted in 14 upregulated ABC genes (Supplementary Figure 1L) and 24 downregulated ABC genes (Supplementary Figure 1M), which are listed in Supplementary Table 1.

### Characteristics of ABC genes

**Fig. 1:**
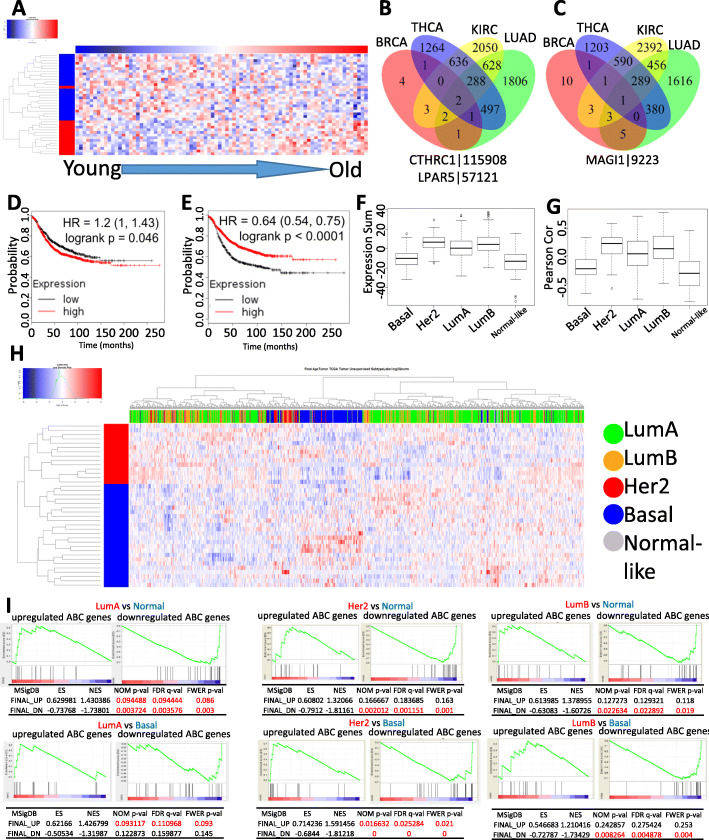
Characterization of 38 ABC genes. **a** Heatmap of the expressions of the ABC genes across the age range. **b** Only CTHRC1 and LPAR5 were upregulated, and **c** MAGI was downregulated in lung adenocarcinoma, thyroid carcinoma, and kidney renal papillary cell carcinoma. **d** The average of the expression (log2 normalized counts) of the 14 upregulated genes was used to group patients. **e**, **f** The average of the expression (log2 normalized counts) of the 24 downregulated genes was used to group patients. The correlation (**f** sum of expression; **g** Pearson’s correlation) between gene expression pattern and subtypes of breast cancer tumor samples. **h** Heatmap of the expression of the 38 ABC genes in different subtypes of breast cancer. The top side bar colors represent subtypes, and the left side bar colors represent the upregulated (red) or downregulated (blue) ABC genes. **i** Gene set enrichment analysis revealed that the 38 ABC genes were more enriched in luminal and Her2 subtypes

These 38 ABC genes clearly showed an age-dependent expression pattern in the 82 adjacent normal samples (Fig. 1a), while such pattern was not observed in those corresponding matched tumor samples (Supplementary Figure 2). Interestingly, most of these 38 genes were not altered in other cancers. Only CTHRC1 and LPAR5 (Fig. 1b) were upregulated and MAGI1 (Fig. 1c) was downregulated in lung adenocarcinoma, thyroid carcinoma, and kidney and renal papillary cell carcinoma in addition to invasive breast cancer when these genes were queried from TCGA database. Importantly, both the higher average expression of the 14 upregulated ABC genes (Fig. 1d) and the lower average expression of the 24 downregulated ABC genes (Fig. 1e) were significantly predictive for poor relapse-free survival in all breast cancer patients from KM plotter dataset [5].

**Fig. 2:**
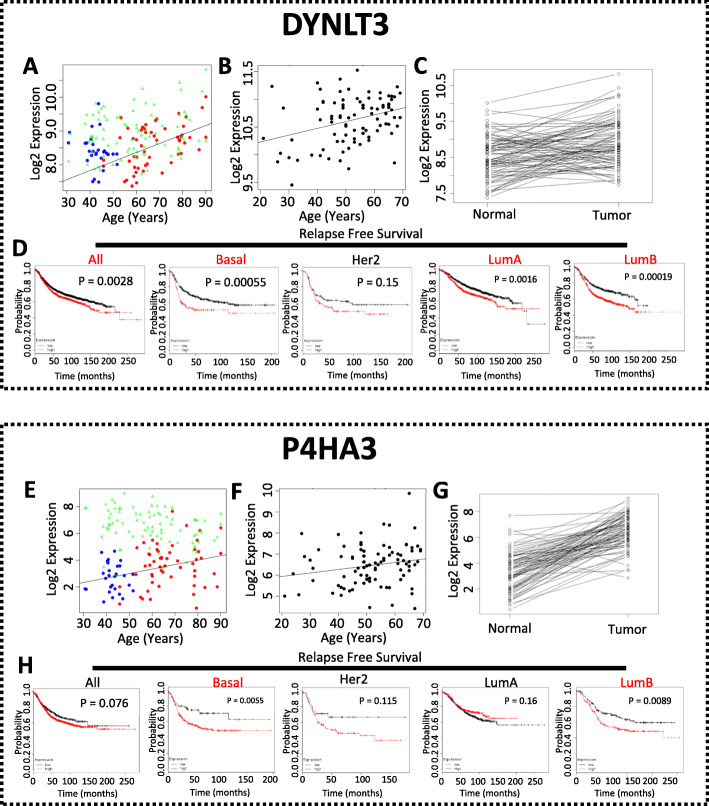
Characteristics of DYNLT3 and P4HA3. **a**, **e** The expression of DYNLT3 or P4HA3 in pre-menopausal normal (blue), post-menopausal normal (red), and tumor (green) samples in TCGA dataset. **b**, **f** The expression of DYNLT3 or P4HA3 in normal tissues in the GTEx dataset. **c**, **g** The paired comparison of DYNLT3 or P4HA3 between matched tumors and normal tissues in TCGA dataset. **d**, **h** The predictive power of DYNLT3 or P4HA3 for relapse-free survival of all and different subtypes of breast cancer patients

Because the expression data in the TCGA dataset were derived from bulk tissue samples and are not cell type-specific, we queried the 38 ABC genes in The Human Protein Atlas (https://www.proteinatlas.org/) for their cell type-specific expression information in breast tissue based on immunohistochemistry (IHC). Interestingly, while eight genes were not detectable by IHC and ten genes have no IHC data, the expression of the remaining twenty genes is either only detectable or higher in breast epithelial cells in comparison with adipocytes suggesting that the 38 ABC genes are mostly expressed by the breast epithelial cells. We next examined the expression pattern of the 38 ABC genes among different subtypes of breast cancer. We developed two metrics: (1) the sum of these 38 ABC gene expression values (Fig. 1f) and (2) the Pearson correlation (Fig. 1g) of these 38 ABC gene expression values based on whether they were up- or downregulated. Both metrics indicated that luminal A and B and Her2 subtypes had higher expressions of the 14 upregulated genes and lower expressions of the 24 downregulated genes compared to triple-negative and normal-like subtypes. This observation is also reflected in the heatmap shown in Fig. 1h. Consistently, the signatures of 14 upregulated genes and 24 downregulated genes were more enriched in luminal A and B and Her2 than basal and normal-like subtypes (Fig. 1I). Strikingly, the majority of these 38 genes did not exhibit an increased number of genetic alterations except CTHRC1 and ETV3L (Supplementary Figure 3, generated from cBioPortal).

**Fig. 3:**
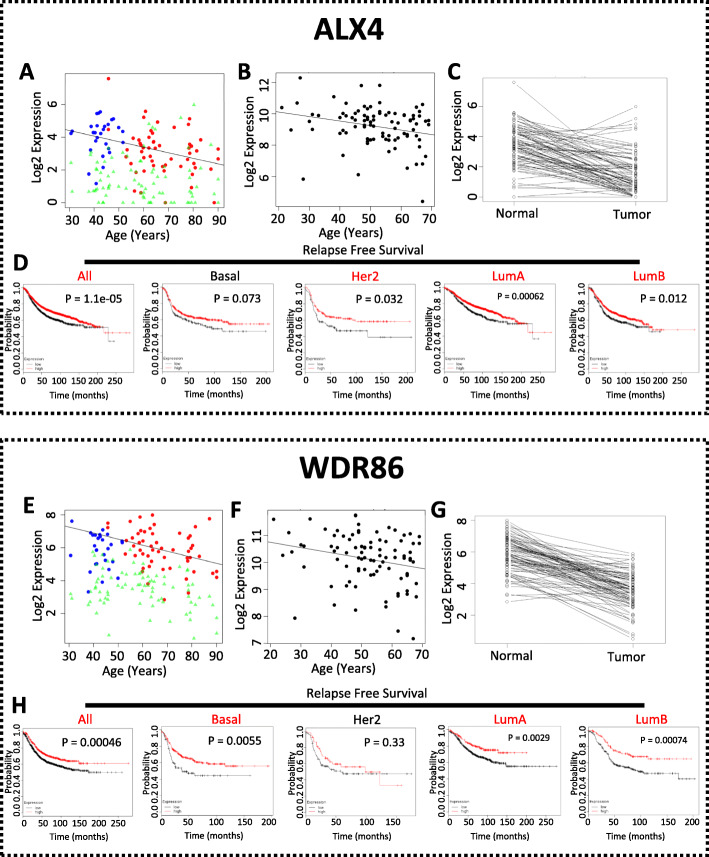
Characteristics of ALX4 and WDR86. **a**, **e** The expression of ALX4 or WDR86 in pre-menopausal normal (blue), post-menopausal normal (red), and tumor (green) samples in TCGA dataset. **b**, **f** The expression of ALX4 or WDR86 in normal tissues in the GTEx dataset. **c**, **g** The paired comparison of ALX4 or WDR86 between matched tumors and normal tissues in TCGA dataset. **d**, **h** The predictive powder of ALX4 or WDR86 for relapse-free survival of all and different subtypes of breast cancer patients

To study whether these ABC genes are promoting or suppressing cancer progression, we developed a scheme to prioritize these genes to a few for experimental validation. Firstly, we conducted a large-scale siRNA screening on the 14 upregulated genes. For each upregulated gene, three siRNAs were transfected into six breast cancer cell lines representing all subtypes of breast cancer (basal, MDA-MB-468; luminal A, MCF7; luminal B, ZR-75-1; Her2, SK-BR-3; claudin-low, Hs578T). From this screening, we found that knockdowns of CLEC3A, CTHRC1, RNASE2, LPAR5, and LRRC15 inhibited the proliferation of all tested cell lines by at least one siRNA (Supplementary Figure 4). Knockdown of DYNLT3 was more effective in inhibiting cell proliferation of MCF7 and SK-BR-3 than ZR-75-1 and Hs578T with little effect on MDA-MB-468. Knockdown of P4HA3 showed a more suppressive effect on MDA-MB-468 and SK-BR-3 than on ZR-75-1 and Hs 578T with a limited effect on MCF7. Knockdown of other upregulated ABC genes including PRR13, EGLN3, HSD11B2, and MATN3 also inhibited the proliferation of various breast cancer cell lines (data not shown). Secondly, to validate the age-association of these genes, we conducted correlation tests between these gene expression and age using 90 samples from the GTEx cohort. We found that 22, 16, and 4 out of the 38 ABC genes showed FDR below 0.5, 0.3, and 0.1, respectively (Supplementary Table 2). The four genes with FDR below 0.1 were CLEC3A, DLK1, DYNLT3, and ETV3L. Because of the large variation in human data and our limited number of samples, we decided to use the cutoff of FDR below 0.5 in combination with other filters discussed here for selecting ABC genes for further study. Thirdly, we examined the predictive prognostic power of individual genes in relapse-free survival of all breast cancer together as well as each subtype separately in a large cohort of breast cancer patients [5]. We found that RNASE2, ARRDC2, PRX, KCNQ4, and DLK1 were significantly predictive in relapse-free survival of all breast cancer patients and in all subtypes whereas EGLN3, DYNLT3, TUBGCP6, HOXA6, and DCHS2 were predictive in all patients, and all subtypes except for those of HER2 subtype, and ENPP6, WDR86, ALX4, CLEC3A, P4HA3, PRR13, CTHRC1, and OTUD7A were predictive in all patients and in at least one subtype of breast cancer patients (data not shown). Using the above filters and reviewing the literature, we selected two upregulated (DYNLT3 and P4HA3, Fig. 2a) and two downregulated (ALX4 and WDR86, Fig. 3a) ABC genes for further functional studies because (1) their functional role in cancer was largely unknown while their expression is significantly upregulated in tumors (Fig. 2c, g) or downregulated (Fig. 3c, g); (2) knockdown of DYNLT3 and P4HA3 by siRNAs inhibited breast cancer cell proliferation (Supplementary Figure 4 and data below); (3) their age-dependent expression pattern was validated in the GTEx dataset (Figs. 2b, f and 3b, f); and (4) they were significantly predictive for relapse-free survival in all breast cancer patients or in patients with certain subtypes of breast cancer (Figs. 2b, h and 3d, h).

### Knockdown of DYNLT3 and P4HA3 reduced breast cancer cell malignancy

**Fig. 4:**
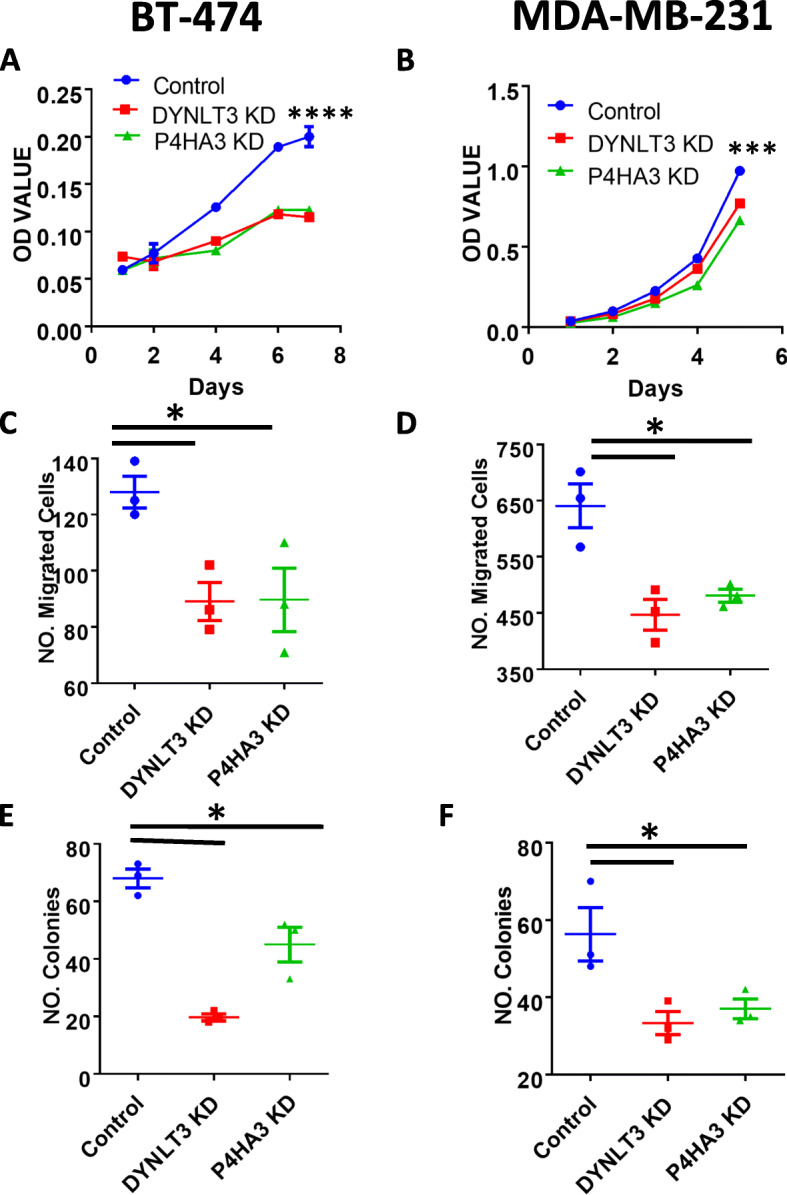
Knockdown of DYNLT3 and P4HA3 reduced the tumor malignancy of BT-474 and MDA-MB-231 cells in vitro*.* Growth of breast cancer cells of BT-474 (**a**) and MDA-MB-231 (**b**). Migration capacity of breast cancer cells of BT-474 (**c**) and MDA-MB-231 (**d**). Colony-forming capacity of breast cancer cells of BT-474 (**e**) and MDA-MB-231 (**f**) in soft agar. Data are presented as the mean ± sem of three measurements. Two-sample *t* tests were used to compare the means of the control and each knockdown group. *P < 0.05; ***P < 0.0005; ****P < 0.0001

To test the potential tumor-promoting roles of the two upregulated ABC genes DYNLT3 and P4HA3, we knocked down their expression in the basal-like breast cancer MDA-MB-231 cells and the luminal B breast cancer BT-474 cells. We chose the BT-474 cell line to represent luminal B subtype model because the 38 ABC genes were significantly enriched in the luminal B subtype. We also included MDA-MB-231 to contrast with BT-474. Both cell lines have tumorigenesis capacity in immune-deficient mice, which allows us to study their potential tumor-promoting role in vivo. Firstly, we confirmed the knockdown of these two genes in the two cell lines by Western blot (Supplementary Figure 5A-D). Next, we examined how their knockdown affected the malignant properties of the two cancer cell lines in vitro. We found that knockdown of either DYNLT3 or P4HA3 significantly reduced the tumor malignant properties in both cell lines, in terms of cell proliferation (Fig. 4a, b), migration (Fig. 4c, d), and anchorage-independent colony formation efficiency in soft agar (Fig. 4e, f). These data indicate that these two upregulated ABC genes are necessary for the malignant properties of the breast cancer cells. It is notable that the extent to which the knockdown of either gene inhibited proliferation of MDA-MB-231 was more moderate than that of BT-474. This could possibly be due to MDA-MB-231 being a faster-growing cell line than BT-474. Therefore, the moderate inhibition in MDA-MB-231 upon knockdown of either DYNLT3 or P4HA3 could be as large as in BT-474. Clearly, further studies are needed to elucidate the differences and similarities of the functions of the two genes in different subtypes of breast cancer cells.

### Knockdown of P4HA3 but not DYNLT3 reduced malignancy in vivo

**Fig. 5:**
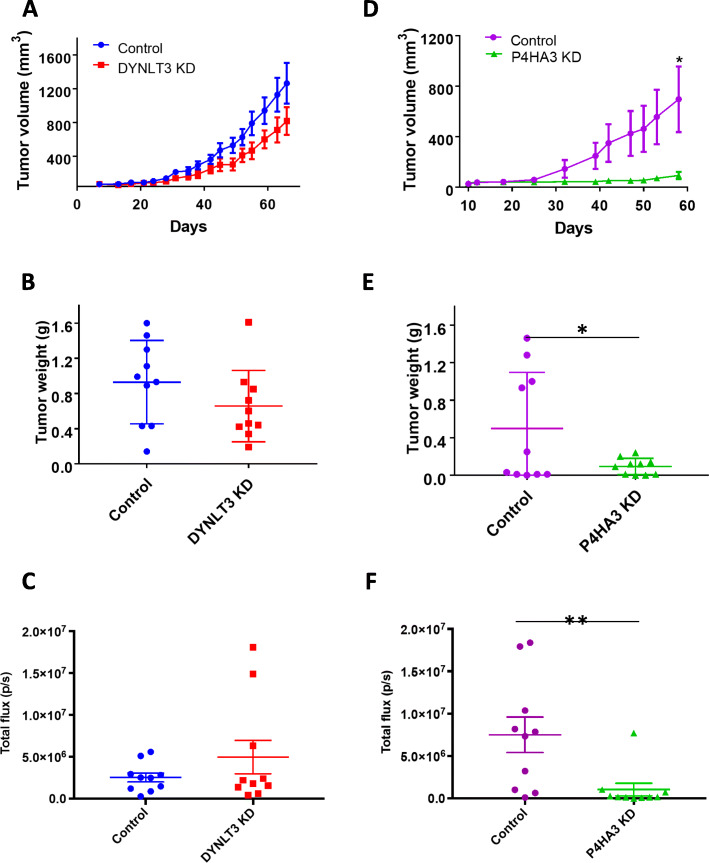
Knockdown of P4HA3 but not DYNLT3 significantly reduced malignancy of BT-474 cell in vivo*.* Comparison of tumor volume over time (**a**, **d**), final tumor weight (**b**, **e**), and total flux from luciferase activity in the lungs (**c**, **f**) between control and DYNLT3 or P4HA3 knockdown BT-474 cell-inoculated mice. Data are presented as the mean ± sem of ten tumors. Two-sample *t* tests were used to analyze the data.*P < 0.05; **P < 0.005

We next examined the roles of DYNLT3 and P4HA3 in tumorigenesis in vivo. The control and DYNLT3 knockdown BT-474 cells expressing ectopic luciferase (Luc) and green fluorescent protein (GFP) were injected subcutaneously into female NOD scid gamma (NSG) mice bilaterally in the flank with 10 mice in each group. In contrast to the significant reduction of the malignant properties in vitro, we observed reduced tumor growth of DYNLT3 knockdown cells but with no statistical significance in comparison with the tumors formed the control cells in terms of tumor volume (Fig. 5a) and tumor weight (Fig. 5b). Consistently, the knockdown of DYNLT3 showed no effect on the extent of lung metastasis as measured with bioluminescence imaging and expressed as the total light flux of luciferase activity in the excised whole lung (Fig. 5c, Supplementary Figure 6). We confirmed the constitutive knockdown of DYNLT3 in the xenograft tumors (Supplementary Figure 7). This appears to indicate that the reduced malignancy due to DYNLT3 knockdown in vitro was somehow compensated under the in vivo condition. Next, to examine whether the reduced in vitro malignancy due to P4HA3 knockdown can be similarly compensated in vivo, we injected the control and P4HA3 knockdown BT-474/Luc-GFP cells subcutaneously in female NSG mice bilaterally in the flank with 10 mice in each group. In contrast to the knockdown of DYNLT3, the knockdown of P4HA3 significantly inhibited tumor progression and metastasis in vivo in terms of tumor volume (Fig. 5d), tumor weight (Fig. 5e), and total flux of luciferase activity in the lungs (Fig. 5f, Supplementary Figure 8). Similarly, we confirmed the constitutive knockdown of P4HA3 in the xenograft tumors (Supplementary Figure 9). Thus, the deficiency of P4HA3, which functions mainly as a mediator of collagen synthesis [8], consistently reduced tumor malignancy both in vitro and in vivo, suggesting that collagen synthesis pathways and extracellular matrix modification is an important regulator of tumor progression.

### Overexpression of ALX4, but not WDR86, reduced breast cancer cell malignancy

**Fig. 6:**
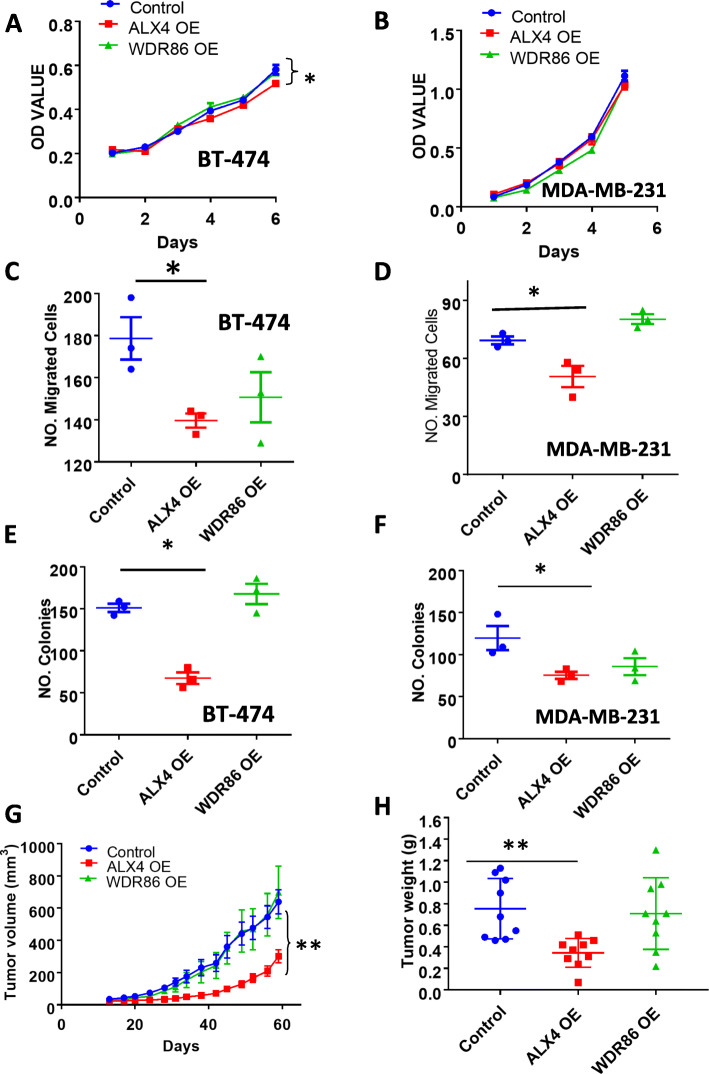
Effect of the overexpression of ALX4 and WDR86 on the malignant properties of BT-474 and MDA-MB-231 cells. Growth of breast cancer cells of BT-474 (**a**) and MDA-MB-231 (**b**). Migration capacity of breast cancer cells of BT-474 (c) and MDA-MB-231 (**d**). Colony-forming capacity of breast cancer cells of BT-474 (**e**) and MDA-MB-231 (**f**). Comparison of tumor volume over time (**g**) and final tumor weight (**h**) between control and ALX4- or WDR86-overexpressing BT-474 cell-inoculated mice. Data are presented as the mean ± sem from three measurements (**a**–**f**) or nine tumors (**g**, **h**). Two-sample *t* tests were used to compare the means of the control and each knockdown group.*P < 0.05; **P < 0.005

To test the function of the two downregulated ABC genes, ALX4 and WDR86, we ectopically introduced their cDNA via a lentiviral vector to increase their expression in the breast cancer cell lines. We found that overexpression of ALX4 moderately, but significantly, inhibited the growth of BT-474 cells (Fig. 6a), but not the growth of MDA-MB-231 cells (Fig. 6b). Nevertheless, the overexpression of ALX4 was found to significantly inhibit migration (Fig. 6c, d) and anchorage-independent colony-forming capacity (Fig. 6e, f) of both BT-474 and MDA-MB-231 cells. Conversely, knockdown of ALX4 in BT-474 cells with a siRNA significantly promoted cell migration in comparison with a control siRNA-transfected BT-474 cells (Supplementary Figure 10). On the other side, overexpression of WDR86 had no effect on these malignant properties of either cell line (Fig. 6a–f). These results suggest that ALX4, but not WDR86, plays a tumor-suppressive role in breast cancer cells, consistent with the reports that ALX4 is downregulated in various cancers [9–11].

To confirm our findings from the in vitro assays, we next examined the effect of overexpression of ALX4 or WDR86 on breast cancer cell growth in a xenograft model in vivo. The control, ALX4-overexpressing, and WDR86-overexpressing BT-474/Luc-GFP cells were injected into NSG mice bilaterally with 9 mice in each group. We found that overexpression of ALX4 significantly inhibited the growth of tumors formed by BT-474 in vivo in terms of both tumor volume (Fig. 6g) and weight (Fig. 6h), supporting our conclusion that ALX4 is tumor-suppressive. In contrast, overexpression of WDR86 did not inhibit tumor progression (Fig. 6g, h), which is consistent with the in vitro data.

## Discussion

Age is a well-known risk factor for breast cancer, and published studies have implicated potential relation between age and breast cancer [12, 13]. Similar to our study, Pirone et al. collected 96 tissue specimens from patients with reduction mammoplasty with age ranging from 14 to 70 years old and studied the transcriptome profile by microarray [14]. The age-associated 802 probes (481 increased, 321 decreased with increasing age) they identified were enriched in biological processes including cancer, immune response, and cellular proliferation. However, only 31 upregulated and 16 downregulated genes overlap between our study and that of Pirone et al. (Supplementary Figure 11 A, B). The small number of overlapping genes could be due to the difference between tumor-adjacent normal tissue and mammoplasty reduction tissue. The latter likely contained more adipose tissue. In addition, the transcriptome profile of the tumor-matched adjacent normal samples in TCGA dataset may be influenced by the presence of cancer [15], which may contribute to the difference between the genes we identified and the age-associated genes by others. Nonetheless, while the age-associated genes we identified may be confounded by the cancer field effect, our main focus was to identify age-associated genes that contribute to tumorigenesis. Thus, in the last step of our transcriptome analysis, we contrasted the age-associated genes with genes differentially expressed in tumor versus their matched adjacent normal tissues resulting in a list of novel ABC genes with predictive power for the disease outcome as well as other unique properties discussed below.

In order to remove the menopause effect in age-related genes, we identified and removed menopause-associated differentially expressed genes from the age-associated genes. However, due to the wide range of age in the post-menopausal patients in the TCGA cohort, such comparison of gene expression between pre- and post-menopausal cohorts may be confounded by age. Therefore, we may lose some important targets in the list of “pure” age-dependent genes (148 upregulated and 189 downregulated genes in Supplementary Figure 1A), resulting in a higher false-negative rate. Menopause has been shown to elevate inflammation response [16, 17]. Aging is associated with chronic inflammation which is also associated with the early development of breast cancer [18]. Upon menopause, systemic hormone level alterations, especially estrogen, lead to the upregulation of estrogen receptor whose activity is critical in breast cancer. Thus, the delineation of the relationship among menopause, aging, and breast cancer development remains an important and challenging topic for further study.

In this study, we identified 38 genes that are both age and breast tumorigenesis related. Among them, fourteen genes are potential tumor-promoting genes that are upregulated during aging while twenty-four of them are potential tumor-suppressive genes that are downregulated during aging. While most of the upregulated genes have previously been shown to be involved in carcinogenesis, most of the downregulated genes have either not been studied for their biological functions or not been implicated in carcinogenesis. For example, the upregulated CLEC3A has been shown highly expressed on the tumor cell surface, and the cleavage of CLEC3A by MMP7 was reported to weaken tumor cell adhesion and migration [19]. The frequently amplified gene CTHRC1 has been reported to be involved in cell proliferation, migration, invasion, and metastasis by regulating multiple signaling pathways including the TGFβ pathway and collagen synthesis pathway [20–23]. The gene HSD11B2 regulated by progesterone, also known as hydroxysteroid (11-beta) dehydrogenase 2, has been frequently studied as a tumor promoter which promotes cancer progression by regulating interconversion of cortisol and cortisone [24–26]. Among the downregulated ABC genes, DLK1 has been shown in many recent studies to function as a tumor suppressor [27, 28]. On the other hand, it was recently shown as non-canonical Notch ligand to promote tumorigenesis by promoting epithelial-mesenchymal transition in ovarian cancer [29]. The gene MAGI1 is short for membrane-associated guanylate kinase inverted 1. Previous studies have suggested its potential tumor suppressor role by interacting with beta-catenin to regulate cell-cell adhesion [30, 31] and by regulating PTEN to inhibit cancer cell migration and invasion [32]. Among the ABC genes that have not been studied in breast cancer, we used the selection criteria described above and selected four novel genes, DYNLT3, P4HA3, ALX4, and WDR86, for further investigation of their functional role in regulating the malignant properties of breast cancer cells for the first time.

With in vitro experiments, we showed that DYNLT3 and P4HA3 promoted breast cancer malignancy. DYNLT3 is short for dynein light chain Tctex-type 3, which is a member of a subclass of dynein light chains. DYNLT3 protein has been reported to form the light chain component of the cytoplasmic dynein motor protein complex [33, 34]. Its paralog, DYNLT1, has been found to promote tumor progression [35–37]. Thus, our current study and the published studies indicate that cytoplasmic dynein motor proteins play a critical role in tumor progression. P4HA3 is short for prolyl 4-hydroxylase subunit alpha 3, which is a component of prolyl 4-hydroxylase involved in collagen synthesis. Its paralog, P4HA1, has been found to promote tumor progression [38, 39]. Alterations in collagen synthesis pathways have been shown in many cancer progressions [40–43]. A recent study showed that P4HA3 appears to be a target effector of the TGFβ pathway mediating the tumor/migration-promoting activity of TGFβ [44]. Consistently, TGFβ1 was found in our study to be significantly upregulated in post-menopausal patients in comparison with pre-menopausal patients in the TCGA dataset (data not shown). On the other side, we showed that overexpression of ALX4, a potential tumor suppressor, significantly inhibited the malignant properties of breast cancer cells. ALX4 is short for aristaless-like homeobox 4, which is a homeobox domain transcription factor. It has been shown in lung cancer [9] and colorectal neoplasia [10] that ALX4 is epigenetically silenced via hypermethylation. Moreover, its expression was previously reported to be significantly downregulated in breast cancer [11]. As we were completing our experiments involving ALX4, Yang and co-workers also reported the tumor-suppressive function of ALX4 in breast cancer cells [45]. These published studies together with ours support the conclusion that ALX4 acts as a putative tumor suppressor in subsets of breast cancer. Lastly, we also studied the extent to which WDR86 could play as a tumor suppressor. WDR86 is a novel gene with little known function. WDR86 is short for WD domain repeat 86. It has been characterized with 4 validated transcription isoforms and 11 predicted isoforms [46]. It is proposed that one of the paralogs of WDR86 is TRAF7, which has been implicated as a tumor suppressor by inducing apoptosis [47, 48]. Therefore, WDR86 may function as a potential tumor suppressor although we did not observe a malignancy-inhibitory activity of WDR86 with the assays we used. Further studies are needed to ascertain the role of WDR86 in breast carcinogenesis. In summary, our current study provides evidence demonstrating tumor-regulatory functions for the three of the four ABC genes. It is possible that some ABC genes such as these three genes may act in concert to regulate breast cancer progression because we found that in about 25% of the 82 cases, they appear co-regulated with a reasonable concurrence score (Supplementary Table 3). However, whether the expression alterations of these genes during aging truly contribute to mammary tumor initiation and progression requires further in vivo experiments using animal models.

One of the potential limitations of our study is that the genes we identified could be limited to the features of 82 patients in TCGA database. Among these 82 patients, there were 45 luminal A subtypes, 17 luminal B subtypes, 13 basal subtypes, 6 Her2 subtypes, and 1 unknown as of the date of analysis. Because of a relatively large variation in human samples, it was impossible to use data of only 45 luminal A subtypes to identify menopause-independent age-associated genes with enough power. Thus, we included all 82 patients for identifying age-associated genes, which could result in a bias toward more luminal-like samples. This may explain why the 38 ABC genes were more positively enriched in luminal A and B and Her2 subtypes of tumors compared to basal and normal-like subtypes. Thus, while the up- or downregulated ABC genes are interestingly associated with poor or favorable patient outcomes, respectively, because of high variation in human samples, more data and studies are needed to confirm the findings in our study.

## Conclusion

Our in silico analyses identified age-associated transcriptome alterations that are involved in tumorigenesis. Specifically, the upregulation of DYNLT3 and P4HA3 as well as the downregulation of ALX4 during aging promote breast cancer progression. These results suggest that transcriptome alterations during aging may contribute to breast tumorigenesis.

## Supplementary information

**Additional file 1: Supplementary Figure 1.** Identification of age and breast cancer associated genes. (A) Workflow of in silico analyses. Volcano plots show the genes expressions in association with age in the matched normal samples from either all 82 breast cancer patients (B) or 54 post-menopausal patients (C). Venn Diagrams show the difference in up-regulated (D) and down-regulated (E) age-associated genes identified from all 82 patients versus from 54 post-menopausal patients. Venn diagrams show the number of genes up-regulated (F) and down-regulated (G) from four models in the tumor-adjacent normal samples comparing post-menopausal to pre-menopausal patients. Venn diagrams show the overlapping and difference in genes that are up-regulated (H) and down-regulated (I) with age and menopause. Venn diagrams show the number of genes up-regulated (J) and down-regulated (K) from four models comparing tumors to their matched normal tissues in pairs. Venn diagrams show 14 up-regulated (L) and 24 down-regulated (M) genes by both age and tumorigenesis.

**Additional file 2: Supplementary Figure 2.** Matched tumor samples do not show age dependent expression. In all 82 matched tumor samples which were ordered by age at diagnosis as indicated by the arrow, no expression pattern of up or down regulated ABC genes was observed. Row side color bar represents genes that were upregulated (red) or downregulated (blue).

**Additional file 3: Supplementary Figure 3.** Scatter Heatmap plot on mutations of ABC genes. CTHRC1 and ETV3L had over 10% alterations in 1074 breast cancer patients (analyzed by cBioPortal, as of August 22nd 2017). Each vertical bar represent one patient. Light gray bars with no red, blue, dark gray or green color represent patients without any genetic alteration in consideration. The color bars at the top depicts patient status, including ER, HER2 and menopause status. The color legends are shown at the bottom.

**Additional file 4: Supplementary Figure 4.** Growth inhibition of breast cancer cell lines by siRNAs targeting selected upregulated ABC genes. Knockdown by siRNAs were performed to study the effect of loss-of-function on 14 up-regulated genes. Knockdowns of seven genes (DYNLT3, P4HA3, CLEC3A, CTHRC1, RNASE2, LPAR5, LRRC15) showed different inhibitory effects on cell proliferation of seven breast cancer cell lines. Three control conditions (green: regular culture; blue: plus transfection reagent; Yellow: plus transfection reagent and a scrambled siRNA) and three siRNAs (black, gray, and red lines) to each gene are included in the experiment.

**Additional file 5: Supplementary Figure 5.** Confirmation of knockdown and overexpression of the depicted gene proteins with Western blotting. Knockdown of DYNLT3 and P4HA3 proteins and overexpression of ALX4 and WDR86 proteins were confirmed in both BT-474 and MDA-MB-231 cell lines with Western blotting.

**Additional file 6: Supplementary Figure 6.** DYNLT3 knockdown in BT-474 cells showed no effect on their lung metastatic potential from subcutaneous tumors in NSG Mice. Completely excised lung tissue from each mouse was placed in the well of 24-well place. Gray images were taken to show the whole lung tissue. Appropriate color scale was overlaid on top of gray images to depict the total flux signal received from luciferase activity. Top two rows are images of ten lungs from the control cell-inoculated mice and bottom two rows are images of ten lungs from the DYNLT3 knockdown cell-inoculated mice.

**Additional file 7: Supplementary Figure 7.** Confirmation of DYNLT3 knockdown in tumors formed by DYNLT3 Knockdown BT-474 cells. Protein expression level of DYNLT3 were measured by Western blotting in the tumors formed by control and DYNLT3 Knockdown BT-474 cells in NSG mice.

**Additional file 8: Supplementary Figure 8.** P4HA3 knockdown in BT-474 cells reduced their lung metastatic potential in NSG mice. Completely excised lung tissue from each mouse was placed in the well of 24-well place. Gray images were taken to show the whole lung tissue. Appropriate color scale was overlaid on top of gray images to depict the total flux signal received from luciferase activity. Top two rows are images of ten lungs from the control cell-inoculated mice and bottom two rows are images of ten lungs from the P4HA3 knockdown cell-inoculated mice.

**Additional file 9: Supplementary Figure 9.** Confirmation of P4HA3 knockdown in tumors formed by P4HA3 Knockdown BT-474 cells. Protein expression level of P4HA3 were measured by Western blotting in the tumors formed by control and P4HA3 Knockdown BT-474 cells in NSG mice.

**Additional file 10: Supplementary Figure 10.** ALX4 KD by siRNA promoted migration of BT-474 cells. (A) Relative gene expression level of ALX4 measured by RT-qPCR in control siRNA or ALX4 siRNA transfected cells. (B) Cell migration was increased upon ALX4 knockdown. Data are presented as the mean ± sem from three measurements. Two-sample t tests were used to analyze the data. **: *P* < 0.005.

**Additional file 11: Supplementary Figure 11.** Comparison to aging genes identified in previous studies. The genes we found up-regulated (A) and down-regulated (B) during aging were compared to those reported by Jason et al. [14]. Similarly, These genes identified in current study were contrasted to the reported common genes up-regulated (C) and down-regulated (D) during aging across rat, mouse and human.

**Additional file 12:Supplementary Table 1.** Summary of Aging Breast Cancer (ABC) Genes.

**Additional file 13: Supplementary Table 2.** Summary of validation of age-associated expression of ABC genes in GTEx Dataset.

**Additional file 14: Supplementary Table 3.** Concurrence score of co-regulation of ALX4, P4HA3 and DYNLT3 in 82 TCGA patients.

## Data Availability

Our results are in part based upon data generated by the TCGA Research Network (https://www.cancer.gov/tcga) and by the GTEx Consortium (www.gtexportal.org/home/).

## Abbreviations

ABC: Age and breast cancer
ANOVA: Analysis of variance
GTEx: Gene to Expression
RNAseq: RNA sequencing
SVA: Surrogate variable analysis
TCGA: The Cancer Genome Atlas

## References

[1] Ferguson NL, Bell J, Heidel R, Lee S, Vanmeter S, Duncan L, Munsey B, Panella T, Orucevic A (2013). Prognostic value of breast cancer subtypes, Ki-67 proliferation index, age, and pathologic tumor characteristics on breast cancer survival in Caucasian women. Breast J.

[2] Hainaut P, Plymoth A (2013). Targeting the hallmarks of cancer: towards a rational approach to next-generation cancer therapy. Curr Opin Oncol.

[3] Hanahan D, Weinberg RA (2011). Hallmarks of cancer: the next generation. Cell.

[4] Lopez-Otin C, Blasco MA, Partridge L, Serrano M, Kroemer G (2013). The hallmarks of aging. Cell.

[5] Gyorffy B, Lanczky A, Eklund AC, Denkert C, Budczies J, Li Q, Szallasi Z (2010). An online survival analysis tool to rapidly assess the effect of 22,277 genes on breast cancer prognosis using microarray data of 1,809 patients. Breast Cancer Res Treat.

[6] Biswas T, Gu X, Yang J, Ellies LG, Sun LZ (2014). Attenuation of TGF-beta signaling supports tumor progression of a mesenchymal-like mammary tumor cell line in a syngeneic murine model. Cancer Lett.

[7] Mishra S, Tai Q, Gu X, Schmitz J, Poullard A, Fajardo RJ, Mahalingam D, Chen X, Zhu X, Sun LZ (2015). Estrogen and estrogen receptor alpha promotes malignancy and osteoblastic tumorigenesis in prostate cancer. Oncotarget.

[8] Luo YF, Xu W, Chen H, Warburton D, Dong R, Qian BP, Selman M, Gauldie J, Kolb M, Shi W (2015). A novel profibrotic mechanism mediated by TGF-stimulated collagen prolyl hydroxylase expression in fibrotic lung mesenchymal cells. J Pathol.

[9] Liu WB, Han F, Du XH, Jiang X, Li YH, Liu Y, Chen HQ, Ao L, Cui ZH, Cao J (2014). Epigenetic silencing of Aristaless-like homeobox-4, a potential tumor suppressor gene associated with lung cancer. Int J Cancer.

[10] Zou H, Harrington JJ, Shire AM, Rego RL, Wang L, Campbell ME, Oberg AL, Ahlquist DA (2007). Highly methylated genes in colorectal neoplasia: implications for screening. Cancer Epidemiol Biomark Prev.

[11] Chang H, Mohabir N, Done S, Hamel PA (2009). Loss of ALX4 expression in epithelial cells and adjacent stromal cells in breast cancer. J Clin Pathol.

[12] Arvold ND, Taghian AG, Niemierko A, Abi Raad RF, Sreedhara M, Nguyen PL, Bellon JR, Wong JS, Smith BL, Harris JR (2011). Age, breast cancer subtype approximation, and local recurrence after breast-conserving therapy. J Clin Oncol.

[13] Yancik R, Wesley MN, Ries LA, Havlik RJ, Edwards BK, Yates JW (2001). Effect of age and comorbidity in postmenopausal breast cancer patients aged 55 years and older. JAMA.

[14] Pirone JR, D’Arcy M, Stewart DA, Hines WC, Johnson M, Gould MN, Yaswen P, Jerry DJ, Smith Schneider S, Troester MA (2012). Age-associated gene expression in normal breast tissue mirrors qualitative age-at-incidence patterns for breast cancer. Cancer Epidemiol Biomark Prev.

[15] Deng G, Lu Y, Zlotnikov G, Thor AD, Smith HS (1996). Loss of heterozygosity in normal tissue adjacent to breast carcinomas. Science.

[16] Iyengar NM, Morris PG, Zhou XK, Gucalp A, Giri D, Harbus MD, Falcone DJ, Krasne MD, Vahdat LT, Subbaramaiah K (2015). Menopause is a determinant of breast adipose inflammation. Cancer Prev Res (Phila).

[17] Iyengar NM, Zhou XK, Gucalp A, Morris PG, Howe LR, Giri DD, Morrow M, Wang H, Pollak M, Jones LW (2016). Systemic correlates of white adipose tissue inflammation in early-stage breast cancer. Clin Cancer Res.

[18] Dong Q, Gao H, Shi Y, Zhang F, Gu X, Wu A, Wang D, Chen Y, Bandyopadhyay A, Yeh IT (2016). Aging is associated with an expansion of CD49fhi mammary stem cells that show a decline in function and increased transformation potential. Aging (Albany NY).

[19] Tsunezumi J, Higashi S, Miyazaki K (2009). Matrilysin (MMP-7) cleaves C-type lectin domain family 3 member A (CLEC3A) on tumor cell surface and modulates its cell adhesion activity. J Cell Biochem.

[20] Pyagay P, Heroult M, Wang Q, Lehnert W, Belden J, Liaw L, Friesel RE, Lindner V (2005). Collagen triple helix repeat containing 1, a novel secreted protein in injured and diseased arteries, inhibits collagen expression and promotes cell migration. Circ Res.

[21] Wang P, Wang YC, Chen XY, Shen ZY, Cao H, Zhang YJ, Yu J, Zhu JD, Lu YY, Fang JY (2012). CTHRC1 is upregulated by promoter demethylation and transforming growth factor-beta1 and may be associated with metastasis in human gastric cancer. Cancer Sci.

[22] Park EH, Kim S, Jo JY, Kim SJ, Hwang Y, Kim JM, Song SY, Lee DK, Koh SS (2013). Collagen triple helix repeat containing-1 promotes pancreatic cancer progression by regulating migration and adhesion of tumor cells. Carcinogenesis.

[23] Kim JH, Baek TH, Yim HS, Kim KH, Jeong SH, Kang HB, Oh SS, Lee HG, Kim JW, Kim KD (2013). Collagen triple helix repeat containing-1 (CTHRC1) expression in invasive ductal carcinoma of the breast: the impact on prognosis and correlation to clinicopathologic features. Pathol Oncol Res.

[24] Subtil-Rodriguez A, Millan-Arino L, Quiles I, Ballare C, Beato M, Jordan A (2008). Progesterone induction of the 11beta-hydroxysteroid dehydrogenase type 2 promoter in breast cancer cells involves coordinated recruitment of STAT5A and progesterone receptor to a distal enhancer and polymerase tracking. Mol Cell Biol.

[25] Zbankova S, Bryndova J, Kment M, Pacha J (2004). Expression of 11beta-hydroxysteroid dehydrogenase types 1 and 2 in colorectal cancer. Cancer Lett.

[26] Zhang MZ, Xu J, Yao B, Yin H, Cai Q, Shrubsole MJ, Chen X, Kon V, Zheng W, Pozzi A (2009). Inhibition of 11beta-hydroxysteroid dehydrogenase type II selectively blocks the tumor COX-2 pathway and suppresses colon carcinogenesis in mice and humans. J Clin Invest.

[27] Kawakami T, Chano T, Minami K, Okabe H, Okada Y, Okamoto K (2006). Imprinted DLK1 is a putative tumor suppressor gene and inactivated by epimutation at the region upstream of GTL2 in human renal cell carcinoma. Hum Mol Genet.

[28] Nueda ML, Naranjo AI, Baladron V, Laborda J (2017). Different expression levels of DLK1 inversely modulate the oncogenic potential of human MDA-MB-231 breast cancer cells through inhibition of NOTCH1 signaling. FASEB J.

[29] Huang CC, Cheng SH, Wu CH, Li WY, Wang JS, Kung ML, Chu TH, Huang ST, Feng CT, Huang SC (2019). Delta-like 1 homologue promotes tumorigenesis and epithelial-mesenchymal transition of ovarian high-grade serous carcinoma through activation of Notch signaling. Oncogene.

[30] Dobrosotskaya IY, James GL (2000). MAGI-1 interacts with beta-catenin and is associated with cell-cell adhesion structures. Biochem Biophys Res Commun.

[31] Gregorc U, Ivanova S, Thomas M, Guccione E, Glaunsinger B, Javier R, Turk V, Banks L, Turk B (2007). Cleavage of MAGI-1, a tight junction PDZ protein, by caspases is an important step for cell-cell detachment in apoptosis. Apoptosis.

[32] Zhang G, Wang Z (2011). MAGI1 inhibits cancer cell migration and invasion of hepatocellular carcinoma via regulating PTEN. Zhong Nan Da Xue Xue Bao Yi Xue Ban.

[33] Lo KW, Kogoy JM, Pfister KK (2007). The DYNLT3 light chain directly links cytoplasmic dynein to a spindle checkpoint protein, Bub3. J Biol Chem.

[34] Wilson MJ, Salata MW, Susalka SJ, Pfister KK (2001). Light chains of mammalian cytoplasmic dynein: identification and characterization of a family of LC8 light chains. Cell Motil Cytoskeleton.

[35] Ochiai K, Watanabe M, Ueki H, Huang P, Fujii Y, Nasu Y, Noguchi H, Hirata T, Sakaguchi M, Huh NH (2011). Tumor suppressor REIC/Dkk-3 interacts with the dynein light chain, Tctex-1. Biochem Biophys Res Commun.

[36] de Wit NJ, Verschuure P, Kappe G, King SM, de Jong WW, van Muijen GN, Boelens WC (2004). Testis-specific human small heat shock protein HSPB9 is a cancer/testis antigen, and potentially interacts with the dynein subunit TCTEL1. Eur J Cell Biol.

[37] Sarma NJ, Yaseen NR (2013). Dynein light chain 1 (DYNLT1) interacts with normal and oncogenic nucleoporins. PLoS One.

[38] Feng G, Shi H, Li J, Yang Z, Fang R, Ye L, Zhang W, Zhang X (2016). MiR-30e suppresses proliferation of hepatoma cells via targeting prolyl 4-hydroxylase subunit alpha-1 (P4HA1) mRNA. Biochem Biophys Res Commun.

[39] Chakravarthi BV, Pathi SS, Goswami MT, Cieslik M, Zheng H, Nallasivam S, Arekapudi SR, Jing X, Siddiqui J, Athanikar J (2014). The miR-124-prolyl hydroxylase P4HA1-MMP1 axis plays a critical role in prostate cancer progression. Oncotarget.

[40] Fang M, Yuan J, Peng C, Li Y (2014). Collagen as a double-edged sword in tumor progression. Tumour Biol.

[41] Phang JM, Liu W, Hancock CN, Fischer JW (2015). Proline metabolism and cancer: emerging links to glutamine and collagen. Curr Opin Clin Nutr Metab Care.

[42] Cechowska-Pasko M, Kretowski R, Bankowski E (2011). Glucose deficiency reduces collagen synthesis in breast cancer MCF7 cells. Cell Biol Int.

[43] Kalluri R (2016). The biology and function of fibroblasts in cancer. Nat Rev Cancer.

[44] Luo Y, Xu W, Chen H, Warburton D, Dong R, Qian B, Selman M, Gauldie J, Kolb M, Shi W (2015). A novel profibrotic mechanism mediated by TGFbeta-stimulated collagen prolyl hydroxylase expression in fibrotic lung mesenchymal cells. J Pathol.

[45] Yang J, Han F, Liu W, Chen H, Hao X, Jiang X, Yin L, Huang Y, Cao J, Zhang H (2017). ALX4, an epigenetically down regulated tumor suppressor, inhibits breast cancer progression by interfering Wnt/beta-catenin pathway. J Exp Clin Cancer Res.

[46] Ota T, Suzuki Y, Nishikawa T, Otsuki T, Sugiyama T, Irie R, Wakamatsu A, Hayashi K, Sato H, Nagai K (2004). Complete sequencing and characterization of 21,243 full-length human cDNAs. Nat Genet.

[47] Scudiero I, Zotti T, Ferravante A, Vessichelli M, Reale C, Masone MC, Leonardi A, Vito P, Stilo R (2012). Tumor necrosis factor (TNF) receptor-associated factor 7 is required for TNFα-induced Jun NH2-terminal kinase activation and promotes cell death by regulating polyubiquitination and lysosomal degradation of c-FLIP protein. J Biol Chem.

[48] Xu LG, Li LY, Shu HB (2004). TRAF7 potentiates MEKK3-induced AP1 and CHOP activation and induces apoptosis. J Biol Chem.

