# Supplementary material for: Age-associated genes in human mammary gland drive human breast cancer progression
Source: Breast Cancer Res. 2020 Jun 15;22:64. doi: 10.1186/s13058-020-01299-2 (PMC7294649; doi:10.1186/s13058-020-01299-2)

Supplementary Figure 2. Matched Tumor Samples Do NOT Show Age Dependent Expression

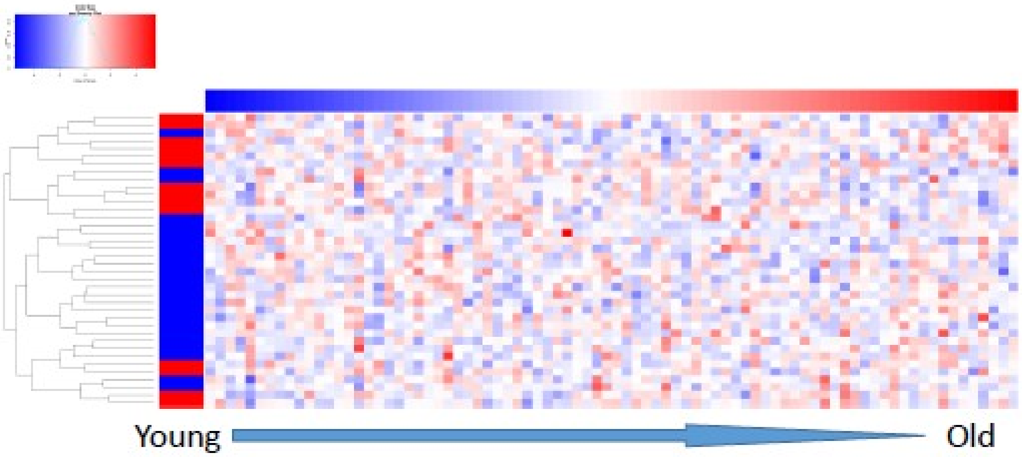

Supplement: Supplementary file 4 [file 13058_2020_1299_MOESM2_ESM.docx]
